# Parent-Reported Otorrhea in Children with Tympanostomy Tubes: Incidence and Predictors

**DOI:** 10.1371/journal.pone.0069062

**Published:** 2013-07-12

**Authors:** Thijs M. A. van Dongen, Geert J. M. G. van der Heijden, Hanneke G. Freling, Roderick P. Venekamp, Anne G. M. Schilder

**Affiliations:** 1 Department of Epidemiology, Julius Center for Health Sciences and Primary Care, University Medical Center Utrecht, Utrecht, The Netherlands; 2 Division Surgical Specialties, Department of Otorhinolaryngology, University Medical Center Utrecht, Utrecht, The Netherlands; 3 ENT Clinical Trials Programme, Ear Institute, Faculty of Brain Sciences, University College London, London, United Kingdom; Charité, Campus Benjamin Franklin, Germany

## Abstract

**Purpose:**

Although common in children with tympanostomy tubes, the current incidence of tympanostomy tube otorrhea (TTO) is uncertain. TTO is generally a sign of otitis media, when middle ear fluid drains through the tube. Predictors for otitis media are therefore suggested to have predictive value for the occurrence of TTO.

**Objective:**

To determine the incidence of TTO and its predictors.

**Methods:**

We performed a cohort study, using a parental web-based questionnaire to retrospectively collect data on TTO episodes and its potential predictors from children younger than 10 years of age with tympanostomy tubes.

**Results:**

Of the 1,184 children included in analyses (total duration of time since tube placement was 768 person years with a mean of 7.8 months per child), 616 children (52%) experienced one or more episodes of TTO. 137 children (12%) had TTO within the calendar month of tube placement. 597 (50%) children had one or more acute TTO episodes (duration <4 weeks) and 46 children (4%) one or more chronic TTO episodes (duration ≥4 weeks). 146 children (12%) experienced recurrent TTO episodes. Accounting for time since tube placement, 67% of children developed one or more TTO episodes in the year following tube placement. Young age, recurrent acute otitis media being the indication for tube placement, a recent history of recurrent upper respiratory tract infections and the presence of older siblings were independently associated with the future occurrence of TTO, and can therefore be seen as predictors for TTO.

**Conclusions:**

Our survey confirms that otorrhea is a common sequela in children with tympanostomy tubes, which occurrence can be predicted by age, medical history and presence of older siblings.

## Introduction

Tympanostomy tube placement is one of the most common surgical procedures performed in children worldwide, with around 50,000 children in the Netherlands, and almost 700,000 in the United States receiving tubes each year. [1], [2] Indications for tympanostomy tubes include prevention of acute otitis media (AOM) recurrences in children with recurrent AOM and restoration of hearing in children with persistent otitis media with effusion (OME). [3] Tympanostomy tube otorrhea (TTO) is a well-known and common sequela in children with tympanostomy tubes. It is generally a sign of otitis media (OM), when middle ear fluid drains through the tube. TTO can be accompanied by foul odor, pain, and fever and can reduce the child’s quality of life. [4] Moreover it may lead to blockage or early extrusion of the tympanostomy tube and hence impact the child’s hearing. As parents generally hope that tympanostomy tubes will solve their child’s middle ear problems, they may be disappointed, or anxious, when their child develops TTO. [5].

Published TTO incidences vary widely and the most recent publications on this topic date from 2001. In that year, a meta-analysis reported an average TTO incidence of 26% based on 23 studies with incidences ranging from 4% up to 68%. [6] A subsequent trial reported a TTO incidence of 75% at 12 months after tube placement in children younger than 3 years. [7] Irrespective of the wide range of reported incidences, changes in health care practice over the last decade, such as development of new OM guidelines and the introduction of pneumococcal vaccination in children, may have changed the incidence of TTO.

For OM, many risk factors have been established such as age, gender, day-care attendance and household smoking. [8], [9] These factors have also been suggested to have predictive value for the occurrence of TTO, but evidence is limited. [10] In addition, the indication for tympanostomy tube placement and frequent water exposure of the ear during swimming or bathing, have been considered as predictors specific for TTO occurrence. [10].

The objectives of the current study are to establish the incidence of TTO in children aged up to 10 years of age with tympanostomy tubes, and to identify predictors for TTO in these children.

## Methods

We designed a cohort study, using a web-based survey to retrospectively collect data on TTO at one point in time from children with tympanostomy tubes. Approval from the Medical Ethics Committee of the University Medical Center Utrecht was obtained.

### Population Characteristics

The survey was conducted among a cohort of 3,559 children up to 10 years of age. They had tympanostomy tubes placed between April 2009 and June 2011 in 18 Dutch general hospitals and two academic hospitals. Children were excluded from the current survey if they had Down’s syndrome, a known immune disorder, cleft lip or palate or if their questionnaire was filled out incompletely.

### Data Collection

Between May and October 2011, a letter was sent to the parents of the children asking them to participate in the survey by filling out a web-based questionnaire regarding potential TTO predictors at the time of the most recent tube placement, TTO occurrence thereafter and time of extrusion of the tympanostomy tube(s) (Appendix S1 and S2). The standardized questionnaire was piloted in a small group of parents of children with tympanostomy tubes and amended based on their responses. It could be filled out at only one point in time. A reminder was sent to the parents who did not complete the questionnaire within 6 weeks after sending the letter.

All children remained under the care of their own family physician and ENT physician throughout our survey. We asked parents if they were willing to fill out a web-based questionnaire and did not attempt to alter local care pathways.

### Data-analysis

We used Rothman’s Episheet (version October 2011) to calculate the incidences. [11] For all other statistical analyses we used SPSS version 17 (SPSS Inc., Chicago, Ill).

#### Time since tympanostomy tube placement

Time since tympanostomy tube placement was defined as starting at the day of the most recent tube placement and ending at the day the web-based questionnaire was filled out. We censored this time period either at the date of tube extrusion as reported by the parents or, when parents were uncertain about presence of tympanostomy tubes, at the day the tubes were last seen in place by a physician. We included all reported TTO episodes during this time period in our analyses.

#### Incidence

We calculated the number of children who had developed 1 or more TTO episodes. Moreover, we assessed the number of children with 2 to 3, or 4 or more episodes of TTO, the numbers of children with early TTO (starting within the calendar month of tube placement), acute TTO (duration <4 weeks), chronic TTO (duration ≥4 weeks) and recurrent TTO (≥3 episodes in 6 months or ≥4 episodes in 12 months), and the proportions of TTO episodes managed by antibiotic(-steroid) eardrops, oral antibiotics or initial observation.

To account for differences in time since tube placement, we assessed incidence densities. We used a Kaplan-Meier curve to depict the time between tube placement and the occurrence of a first TTO episode in the first 12 months after tube placement. We also assessed the median duration between the most recent tube placement and onset of the first TTO episode in children developing TTO, and the TTO incidence rate in all included children.

#### Predictors

We selected candidate predictors based on their suggested or shown association with OM or TTO in the literature (see table 1 for definitions).[7]–[10], [12]–[15], [17], [18] First, we assessed the relation between each of the candidate predictors and our main outcome (one or more episodes of TTO in the time period since tympanostomy tube placement). To account for differences in time since tube placement we used Cox regression analyses using occurrence of the first TTO episode as the outcome. Second, to determine independent predictors, we also performed a multivariable Cox regression analysis. For this analysis, we did not select predictors based on the outcomes of univariable analyses, but included all putative indicators, and used a backward elimination procedure with a cutoff value of p<0.05 to identify independent predictors. In this, we followed the rule of thumb of a minimum of 10 events for each predictor to be included in the multivariable Cox regression analysis. [16] All results were expressed as hazard ratios (HR) with 95% confidence intervals (CI). We dichotomized age (<4 years/≥4 years) for the univariable analysis, but because of potential loss of information we included age as a continuous variable in the multivariable analysis. Guided by the outcomes of the multivariable Cox regression analysis, we calculated the absolute risk (incidence) of TTO in children grouped according to the presence of the independent predictors.

**Table 1:** Univariable and multivariable Cox regression analysis of potential predictors for developing otorrhea in children with tympanostomy tubes.

Potential predictors		≥1 episode of TTO, n (%)		Univariable	Multivariable^i^	
		Yes (n = 616)	No (n = 568)	HR (95% CI)	HR (95% CI)	Coefficient (SE)
Age	<4 years	305 (49.5)	201 (35.4)	1.00	–	–
	≥4 years	311 (50.5)	367 (64.6)	0.72 (0.62; 0.85)	–	–
Age per year increase		–	–	0.91 (0.87; 0.94)	0.95 (0.91; 0.98)	−0.06 (0.02)
Male gender		360 (58.4)	325 (57.2)	1.08 (0.92; 1.27)	NS	NA
Indication for tube placement^ii^	Recurrent acute otitis media	319 (51.8)	204 (35.9)	1.48 (1.26; 1.73)	1.26 (1.06; 1.49)	0.23 (0.09)
	Chronic otitis media with effusion	291 (47.2)	356 (62.7)	0.69 (0.59; 0.80)	NS	NA
Previous tube placement		228 (37.0)	281 (49.5)	0.83 (0.70; 0.97)	NS	NA
Previous ENT surgery	Adenoidectomy	347 (56.3)	279 (49.1)	1.22 (1.04; 1.44)	NS	NA
	Tonsillectomy	130 (21.1)	121 (21.3)	0.99 (0.81; 1.20)	NS	NA
≥6 URTIs in past year		282 (45.8)	183 (32.2)	1.59 (1.35; 1.86)	1.38 (1.17; 1.63)	0.32 (0.09)
Atopy^iii^		199 (32.3)	152 (26.8)	1.12 (0.95; 1.33)	NS	NA
Water exposure		430 (69.8)	415 (73.1)	0.83 (0.70; 0.99)	NS	NA
Attending day-care/school^iv^		282 (92.5)	190 (94.5)	0.79 (0.51; 1.20)	NS	NA
Smoking in household		32 (5.2)	33 (5.8)	0.91 (0.64; 1.30)	NS	NA
Low maternal education level		65 (10.6)	55 (9.7)	1.09 (0.84; 1.41)	NS	NA
Overweight^v^		38 (6.2)	36 (6.3)	0.94 (0.68; 1.31)	NS	NA
≥2 siblings		200 (32.5)	172 (30.3)	1.06 (0.90; 1.26)	NS	NA
Older siblings		363 (58.9)	285 (50.2)	1.29 (1.10; 1.51)	1.21 (1.03; 1.42)	0.19 (0.08)
Family history	Middle ear infections in parents	303 (49.2)	280 (49.3)	1.10 (0.94; 1.29)	NS	NA
	Middle ear infections in siblings^vi^	231 (44.0)	241 (46.5)	1.03 (0.86; 1.22)	NS	NA
	Allergy in parents	324 (52.6)	325 (57.2)	0.92 (0.78; 1.07)	NS	NA
	Allergy in siblings^vi^	168 (32.0)	152 (29.4)	1.08 (0.90; 1.30)	NS	NA
Gestational age <37 weeks		75 (12.2)	68 (12.0)	0.99 (0.78; 1.26)	NS	NA
Birth weight <2500 grams		44 (7.1)	41 (7.2)	1.09 (0.80; 1.48)	NS	NA
Breastfed for >3 months		305 (49.5)	277 (48.8)	0.97 (0.83; 1.13)	NS	NA
Pacifier use in previous year		133 (21.6)	79 (13.9)	1.43 (1.18; 1.73)	NS	NA
PCV7-vaccination		339 (55.0)	251 (44.2)	1.42 (1.21; 1.67)	NS	NA

TTO = tympanostomy tube otorrhea; n = number; HR = hazard ratio; CI = confidence interval; SE = standard error; i = only results presented for independent predictors with p<0.05; NS = not significant; NA = not applicable; ii = 14 children for other indications; ENT = ear, nose and throat; URTI = upper respiratory tract infection; iii = diagnosis of allergic rhinitis, asthma, eczema or food allergy; iv = univariable analysis only including children younger than 4 years, school is mandatory for children 4 years and over; v = BMI was categorized into underweight or normal weight versus overweight according to the World Health Organization standards, corrected for age and gender. [24], [25]; vi = univariable analysis only including 1045 children with siblings; PCV-7 = 7-valent pneumococcal vaccine.

## Results

Parents of 3,559 children who had tympanostomy tubes placed by their local ENT physician were approached and between May and December 2011 we received questionnaires of 1,322 (37%) children (Appendix S3). Of these 1,322 children, we excluded 138 from analysis: 9 with cleft lip or palate, 4 with a known immunodeficiency, 4 with Down’s syndrome, 109 whose questionnaires were not completed and 12 children whose parents had reported incorrect dates making it impossible to calculate the time period since tympanostomy tube placement.

At tube placement, the mean age of the 1,184 included children was 4.4 years (SD: 2.3) and 58% were boys. The total time between tube placement and the survey was 768 person years with a mean of 7.8 months (SD: 5.7, range: 0.3 to 34.0) and a median of 6.4 months (interquartile range: 7.9) per child. Other baseline characteristics of the included children are presented in table 1.

### Incidence

A total of 616 (52.0%) of the children experienced one or more episodes of TTO (table 2). 137 children (11.6%) had otorrhea within the calendar month of tube placement. 597 (50.4%) children had one or more acute TTO episodes with a duration below 4 weeks and 46 (3.9%) one or more chronic TTO episodes (duration ≥4 weeks). 146 (12.3%) of the children experienced recurrent episodes of TTO. 60.5% of the reported TTO episodes had been treated with antibiotic(-steroid) eardrops, 12.9% with oral antibiotics and 36.1% had been managed with initial observation (total exceeds 100% because treatments are not mutually exclusive).

**Table 2:** Incidence of tympanostomy tube otorrhea.

Types of TTO^*^		Children (n = 1,184)	
		n	% (95% CI)
Unspecified			
	1 or more episodes	616	52.0 (49.2; 54.9)
	2 to 3 episodes	213	18.0 (15.9; 20.3)
	4 or more episodes	102	8.6 (7.1; 10.3)
Early		137	11.6 (9.8; 13.5)
Acute		597	50.4 (47.6; 53.3)
Chronic		46	3.9 (2.9; 5.1)
Recurrent		146	12.3 (10.6; 14.3)

TTO = tympanostomy tube otorrhea; * Unspecified = any type of TTO, early = starting within the calendar month of tube placement, acute = duration <4 weeks, chronic = duration ≥4 weeks; recurrent = ≥3 episodes in 6 months or ≥4 episodes in 12 months; n = number; CI = confidence interval.

**Figure 1:**
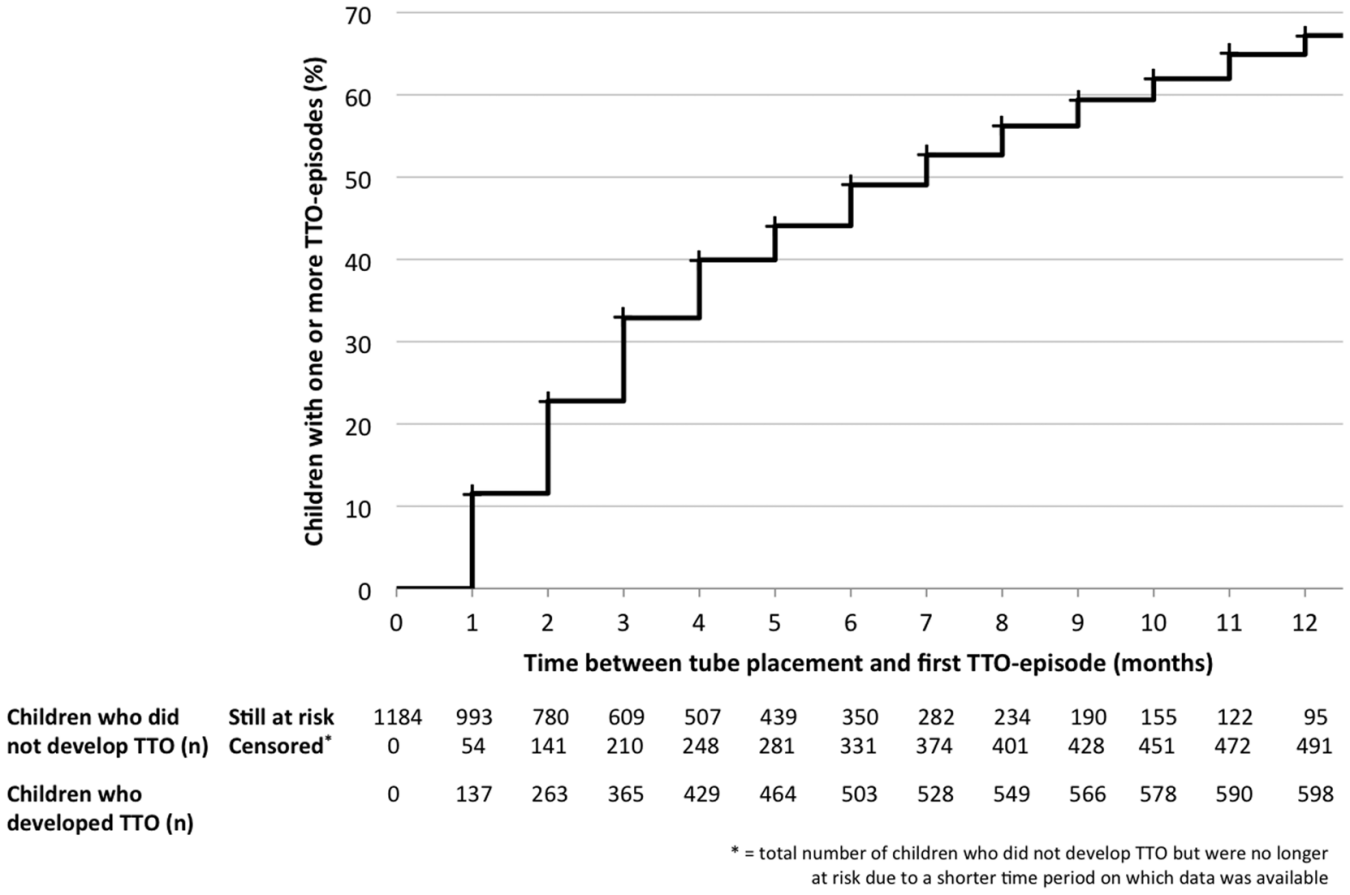
Kaplan-Meier curve for the duration between tube placement and the occurrence of a first TTO episode.

Figure 1 shows the Kaplan-Meier curve of the time between tube placement and the occurrence of a first TTO episode. It demonstrates that at 6 months, 49.1% of the children had developed one or more episodes of TTO. At 12 months this percentage is 67.2%. In the children who experienced TTO, the median time between tube placement and onset of the first episode was 2 months (interquartile range: 3). The TTO incidence rate in our study population was 1.8 (95% CI: 1.7 to 1.9) episodes per person year.

### Predictors

The results of the univariable analyses are presented in table 1. When accounted for differences in time since tympanostomy tube placement and dependency between predictors, age (per year increase: HR = 0.95; 95% CI: 0.91 to 0.98), the indication for tube placement being recurrent acute OM (HR = 1.26; 95% CI: 1.06 to 1.49), a history of 6 or more upper respiratory tract infections in the past year (HR = 1.38; 95% CI: 1.17 to 1.63) and having older siblings (HR = 1.21; 95% CI: 1.03 to 1.42) were found to be independent predictors for the occurrence of otorrhea in children with tympanostomy tubes (see table 1). Table 3 gives an indication of the risk (incidence) of TTO in children according to the presence of these independent predictors (except age): the risk of TTO ranged from 38.1% in children without any of these predictors up to 67.5% in children with all predictors present at tympanostomy tube placement.

**Table 3:** Incidence of tympanostomy tube otorrhea according to presence of independent predictors.

Independent predictors present, n*	≥1 episode of TTO		Total children, n
	n	% (95% CI)	
0	85	38.1 (31.9; 44.6)	223
1	210	46.5 (41.9; 51.1)	452
2	209	60.9 (55.7; 66.0)	343
3	112	67.5 (60.1; 74.3)	166

* recurrent acute otitis media as the indication for tube placement; ≥6 upper respiratory tract infections in past year; presence of older siblings. The above risks are derived from a study population with a mean age of 4.4 years and will be lower in older children and higher in younger children.

## Discussion

In this cohort study of children younger than 10 years of age with tympanostomy tubes, 67% experienced one or more episodes of otorrhea in the year after tube placement. Young age, recurrent acute OM being the indication for tube placement, a recent history of recurrent upper respiratory tract infections and the presence of older siblings are independently associated with the future occurrence of TTO.

This is one of the largest studies on the incidence of TTO. The TTO incidence ascertained in our survey is higher than reported by Kay et al. in 2001. [6] In their meta-analysis they found a wide range of incidences as reported in the different studies, which they explained by differences in study design. In our population 22% of parents contacted the ENT physician, and 17% their family physician every time their child developed TTO (data not shown). This indicates that observational studies relying on medical records are likely to underreport TTO incidence. Clinical trials on the other hand report much higher TTO incidences, as they may include asymptomatic and subclinical episodes. [7] We believe that our parent-reported observational study provides a good estimate of the TTO incidence in children with tympanostomy tubes.

To our knowledge, this is the first study establishing the associations between a comprehensive set of potential predictors and future occurrence of TTO. Previous studies on these associations often used univariable analyses or included a small selection of predictors.[7], [12]–[14], [17], [18] To account for dependencies between predictors, we have performed a multivariable analysis. Because all included children have a history of OM, the absolute hazard ratios, which can also be interpreted as relative risks, are small. The TTO incidence is however considerably higher in children with more independent predictors present at the time of tube placement than those with fewer of these predictors. Our results are consistent with those of Debruyne et al. who labeled age and a history of recurrent acute OM as predictors for TTO, and those of Gates et al. who suggested an association between recurrent upper respiratory tract infections and the occurrence of TTO. [13], [14] A potential TTO-specific predictor is frequent water exposure by bathing or swimming; we however did not find any association. Although pneumococcal vaccination was believed to reduce OM incidence, a recent review suggests that its effect on OM incidence is only marginal. [9], [10], [19] A first glance at our univariable analysis suggests that pneumococcal vaccination may increase the risk of a future occurrence of TTO, however this is easily explained by the fact that all young children in our survey, born since 2006 when pneumococcal vaccination was introduced in The Netherlands, have been vaccinated and the older children have not. Our multivariable analysis revealed no association between pneumococcal vaccination and occurrence of TTO. The surgical rate of tympanostomy tube placement is high in The Netherlands, suggesting that our results may not be generalizable to countries that have a different study domain through use of more stringent criteria for tube placement. [20].

Some aspects of our study deserve further consideration. First, we relied on parental diagnosis of TTO. We previously showed that during follow-up after a physician diagnosis of otorrhea (n = 291 children), there was a high level of agreement between parents and physicians in the assessment of persisting ear discharge. [21] Second, although so far no trials have assessed the long-term effects of treatment for acute TTO, treatment may influence persistence or recurrence of TTO. We therefore provide information on the proportions of TTO episodes treated with antibiotic(-steroid) eardrops, oral antibiotics and initial observation and emphasize that throughout our survey children remained under the care of their local family physician and ENT physician. Third, non-response bias may have affected our results. We explored this by comparing demographics, i.e. age and gender, of responders and non-responders. In addition, we compared TTO incidences as recorded in the medical records of a 10% sample of all responders (n = 144) with those of an equal number of non-responders. Although this does not rule out non-response bias, we did not find differences between these groups for both comparisons (data not shown). Fourth, we collected data on previous TTO episodes using survey methods, whereby recall may have contributed to inaccuracy of our incidence estimates. To address this, we asked for the calendar month and year of TTO episodes rather than the actual day of onset. Our study design therefore allows us to approximate the incidence of early TTO, defined as starting within the calendar month of tube placement. It does however not allow us to determine the incidence of early postoperative TTO, defined as starting within 2 weeks after tube placement. Previous studies comparing parental report of OM with diagnoses recorded in medical records have shown that OM frequency is most prone to bias and the longer the time since OM occurrence, the larger the inaccuracy of recall. [22], [23] We therefore used presence of one or more episodes of TTO as outcome of our Cox regression analyses and Kaplan-Meier curve, rather than the absolute number of episodes. Also, most children in our survey had their tympanostomy tubes placed in the past year, reducing the time since potential TTO occurrence. In addition, as reported above we compared a random sample of medical records with the completed questionnaires of these children and checked the accuracy of verifiable data. We found a high correspondence between the questionnaires and the medical records with regard to patient characteristics, date and number of tympanostomy tube placements and previous ENT surgery (data not shown).

## Conclusion

Our survey confirms that otorrhea is a common sequela in children with tympanostomy tubes: more than half of these children develop at least one episode, in particular young children with older siblings, a recent history of recurrent upper respiratory tract infections and recurrent acute otitis media being the indication for tube placement.

## Supporting Information

Appendix S1 (DOCX)

Appendix S2 (PDF)

Appendix S3 (PDF)
