# Supplementary material for: Parent-Reported Otorrhea in Children with Tympanostomy Tubes: Incidence and Predictors
Source: PLoS One. 2013 Jul 12;8(7):e69062. doi: 10.1371/journal.pone.0069062 (PMC3709928; doi:10.1371/journal.pone.0069062)
Supplement: Appendix S1 — (DOCX) [file pone.0069062.s001.docx]

**Appendix S1**

Parental questionnaire in Dutch, and translated in English.

**Questionnaire (Dutch):**

*Bij eventuele problemen tijdens het invullen van de vragenlijst kunt u contact opnemen met: tel: …… of email: ……*

**Trommelvliesbuisjes**

1. Wat is de geboortedatum van uw kind over wie u deze vragenlijst invult?
   1. dd/mm/jj
2. Hoe vaak zijn in totaal trommelvliesbuisjes bij hem/haar geplaatst (inclusief deze buisjes)?
   1. aantal
3. Wanneer zijn bij hem/haar voor het laatst trommelvliesbuisje(s) geplaatst?
   1. dd/mm/jj
4. Zitten één of beide buisjes nu nog in de oren van uw kind?
   1. ja
   2. nee
      1. wanneer zijn de buisjes uit de oren gegroeid?
         - mm/jj
         - weet niet, de laatste keer dat een arts ze nog in de oren heeft gezien was:
           - mm/jj
   3. weet niet, de laatste keer dat een arts ze nog in de oren heeft gezien was:
      1. mm/jj
5. Wat was volgens de KNO-arts de reden voor het plaatsen van deze trommelvliesbuisjes (meerdere antwoorden mogelijk)?
   1. herhaalde acute middenooroorontstekingen
   2. vocht achter het trommelvlies en niet goed horen
   3. anders, namelijk: …

**Looporen**

*De volgende vragen gaan over looporen in de tijd dat uw zoon/dochter voor het laatst trommelvliesbuisjes had. We spreken van een loopoor als er vocht uit het oor loopt. Dit kan helder vocht zijn, maar ook pus of bloederig vocht.*

1. Hoe vaak heeft uw kind een loopoor gehad terwijl het trommelvliesbuisje nog in het oor zat?
   1. aantal
      1. wanneer was dit en hoe lang duurde het?
         - mm/jj
         - korter dan 2 weken / langer dan 2 weken maar korter dan 4 weken / langer dan 4 weken
      2. welke behandeling(en) heeft uw kind hiervoor gehad? (Het kan zijn dat hij/zij meerdere behandelingen behandelingen heeft gehad, u kunt daarom meerdere opties aanvinken)?
         - we hebben afgewacht of het vanzelf overging
         - oordruppels
         - antibioticumdrankje of tabletten
         - anders, namelijk: …
      3. nam u elke keer contact op met uw arts als uw kind een loopoor kreeg?
         - ja, elke keer met de KNO-arts
         - ja, elke keer met de huisarts
         - ja, maar de ene keer met de huisarts en de andere keer met de KNO-arts
         - nee, ik nam niet bij elk loopoor contact op met mijn arts
2. Welke behandeling zou u kiezen als uw kind een loopoor krijgt terwijl hij/zij trommelvliesbuisjes heeft?
   1. oordruppels
   2. antibioticumdrankje of tabletten
   3. ik zou het liefst afwachten of het vanzelf overgaat

**Andere medische problemen:**

1. Heeft uw kind, voordat de laatste keer trommelvliesbuisjes zijn geplaatst, ook nog andere KNO-operaties ondergaan?
   1. neusamandel geknipt
   2. keelamandelen geknipt
   3. andere operaties aan keel, neus of oren?
2. Hoe vaak is uw kind verkouden (snotneus, hoesten, keelpijn) geweest in het jaar voorafgaand aan het plaatsen van de trommelvliesbuisjes?
   1. minder dan 6 keer
   2. 6 keer of vaker
3. Heeft een arts ooit een van de volgende aandoeningen bij hem/haar vastgesteld?
   1. hooikoorts of allergische verkoudheid; j/n
   2. astma of astmatische bronchitis (hoesten, piepende of zagende ademhaling); j/n
   3. dauwworm of eczeem; j/n
   4. voedselallergie; j/n
   5. Downsyndroom; j/n
   6. lip-/kaak-/gehemeltespleet; j/n
   7. afweerstoornis; j/n
   8. anders namelijk: …

**Over uw zoon/dochter**

1. Na hoeveel weken zwangerschap is hij/zij geboren?
   1. na minder dan 32 weken
   2. na 32-37 weken
   3. na meer dan 37 weken
2. Wat was zijn/haar geboortegewicht?
   1. minder dan 1500 gram
   2. 1500 – 2500 gram
   3. 2500 – 4000 gram
   4. meer dan 4000 gram
3. Heeft uw kind als baby borstvoeding gekregen?
   1. ja
      1. hoeveel maanden heeft hij/zij alleen borstvoeding gekregen?
         - aantal
      2. hoeveel maanden heeft hij/zij borstvoeding in combinatie met flesvoeding gekregen?
         - aantal
   2. nee
4. Heeft uw kind alle inentingen volgens het Rijksvaccinatieprogramma gekregen?
   1. ja
   2. nee
      1. heeft uw kind de pneumokokkenvaccinatie gehad?
         - ja
         - nee
5. Wat is nu zijn/haar gewicht*?*
   1. aantal (kilogram)
6. Wat is nu zijn/haar lengte?
   1. aantal (centimeter)
7. Ging uw kind naar een kinderdagverblijf, peuterspeelzaal of de basisschool in het jaar voorafgaand aan het plaatsen van de laatste trommelvliesbuisjes?
   1. ja, kinderdagverblijf of peuterspeelzaal
   2. ja, school
   3. ja, allebei (kinderdagverblijf/peuterspeelzaal en school)
   4. nee
8. Zat uw kind op zwemles, of ging hij/zij regelmatig zwemmen in een zwembad, meer of zee in het jaar voor de laatste trommelvliesbuisjes werden geplaatst?
   1. ja
   2. nee
      1. ging uw kind wel vaak met zijn/haar hoofd onder water bij het in bad gaan?
         - ja
         - nee
9. Gebruikte uw kind oordopjes bij het zwemmen en/of in bad gaan?
   1. ja, bij het zwemmen
   2. ja, bij het in bad gaan
   3. ja, bij zowel het zwemmen als in bad gaan
   4. nee
10. Gebruikte uw kind een speen in het jaar voordat de laatste buisjes werden geplaatst?
    1. ja
    2. nee

**Over uw gezin**

1. Hoeveel broers of zussen heeft uw kind?
   1. [aantal] oudere broers of zussen
   2. [aantal] jongere broers of zussen
   3. geen broers/zussen
2. Hebben 1 of meer van de overige gezinsleden van uw kind middenoorproblemen (acute middenoorontstekingen of niet goed horen door vocht achter het trommelvlies) gehad?
   1. vader/moeder
      1. ja
      2. nee
   2. broer(s)/zus(sen)
      1. ja
      2. nee
      3. niet van toepassing
3. Is ooit bij een van de andere gezinsleden (ouders, broers, zussen van uw kind) door een arts astma, hooikoorts, eczeem of voedselallergie vastgesteld?
   1. vader/moeder
      1. ja
      2. nee
   2. broer(s)/zus(sen)
      1. ja
      2. nee
4. Toen de laatste keer buisjes werden geplaatst bij uw kind, werd er toen in uw huis gerookt?
   1. ja
      1. hoeveel sigaretten/sigaren worden er in totaal door een of meerdere bewoners per dag in huis gerookt?
         - aantal
   2. nee
5. Wat is de hoogst voltooide opleiding van:
   1. vader
      1. geen opleiding
      2. lagere school
      3. lager voortgezet onderwijs (VMBO, VBO, MAVO, LBO, LTS, VGLO)
      4. hoger voortgezet onderwijs (MMS, HBS, HAVO, VWO)
      5. middelbaar beroeps onderwijs (MBO, MTS, MEAO)
      6. hoger beroeps onderwijs (HBO, HTS) of universiteit
   2. moeder
      1. geen opleiding
      2. lagere school
      3. lager voortgezet onderwijs (VMBO, VBO, MAVO, LBO, LTS, VGLO)
      4. hoger voortgezet onderwijs (MMS, HBS, HAVO, VWO)
      5. middelbaar beroeps onderwijs (MBO, MTS, MEAO)
      6. hoger beroeps onderwijs (HBO, HTS) of universiteit

**Questionnaire (English translation)**

*If you encounter any problems when filling out this questionnaire, please contact us by phone (Telephone number : …) or by email (Email address: …).*

**Tympanostomy tubes**

1. Please would you fill out this questionnaire about your son/daughter with tympanostomy tubes; what is his/her date of birth?
   1. dd/mm/yy
2. On how many different occasions were tympanostomy tube(s) placed in his/her eardrum(s) (including the current tube(s))?
   1. number
3. When was the most recent tympanostomy tube placement?
   1. dd/mm/yy
4. Are the tube(s) still present in his/her eardrum(s)?
   1. yes
   2. no
      1. when did the tubes grow out?
         - mm/yy
         - I’m not sure; the most recent occasion on which a physician has seen the tubes in the eardrum(s) was:
           - mm/yy
   3. I’m not sure; the last occasion on which a physician has seen the tubes in the eardrum(s) was:
      1. mm/yy
5. What was the reason for the most recent tympanostomy tube placement, according to the ENT surgeon (multiple answers possible)?
   1. recurrent acute middle ear infections
   2. reduced hearing and/or fluid behind the eardrum
   3. other reason, namely: …

**Ear discharge**

*The following questions regard episodes of ear discharge during the time period when the most recent tympanostomy tubes were present in the eardrum(s). Ear discharge is fluid that drains from the ear. This fluid can be clear, but can also be bloody or pus-like.*

1. How many times did your child have episodes of ear discharge while the most recent tympanostomy tubes were in place?
   1. number
      1. when did it occur and how long did it last?
         - mm/yy
         - shorter than 2 weeks / between 2 and 4 weeks / longer than 4 weeks
      2. what treatment did your child receive for the ear discharge (multiple answers possible)?
         - none, we waited until it spontaneously resolved
         - eardrops
         - oral antibiotics (by mouth)
         - other treatment, namely: …
      3. did you always contact a physician when your child developed an episode of ear discharge?
         - yes, we contacted the ENT surgeon for each episode
         - yes, we contacted the family physician for each episode
         - yes, we contacted the family physician and/or ENT surgeon on different occasions
         - no, we did not always contact a physician
2. Which treatment do you prefer for your child when he/she develops ear discharge while tympanostomy tubes are present in the eardrum?
   1. waiting until it resolves by itself
   2. eardrops
   3. oral antibiotics (by mouth)

**Medical history**

1. Did your child have any other ENT surgery before the most recent tympanostomy tube placement (multiple answers possible)?
   1. removal of the adenoids
   2. removal of the tonsils
   3. other surgery on ear(s), nose or throat, namely: …
2. How many times did your child develop a cold (runny nose, cough, sore throat) in the year before the most recent tympanostomy tube placement?
   1. less than 6 times
   2. 6 times or more
3. Has your child ever been diagnosed with the following diseases by their physician?
   1. hay fever or allergic rhinitis; y/n
   2. asthma or asthmatic bronchitis; y/n
   3. (atopic) eczema; y/n
   4. food allergy; y/n
   5. Down’s syndrome; y/n
   6. cleft lip/palate; y/n
   7. immune disorder; y/n
   8. other disease, namely: …

**About your son/daughter**

1. After how many weeks gestation was your child born?
   1. after less than 32 weeks
   2. after 32 – 37 weeks
   3. after more than 37 weeks
2. What was his/her birth weight?
   1. less than 1500 grams
   2. 1500 – 2500 grams
   3. 2500 – 4000 grams
   4. more than 4000 grams
3. Was your child breastfed?
   1. yes
      1. how many months was he/she solely breastfed?
         - number
      2. how many months was he/she breastfed in combination with bottle feeding?
         - number
   2. no
4. Was your child vaccinated according to the routine National Vaccination Program?
   1. yes
   2. no
      1. did your child receive the pneumococcal vaccination?
         - yes
         - no
5. What is his/her current weight?
   1. number (kilograms)
6. What is his/her current length/height?
   1. number (centimeters)
7. Did your child attend daycare or go to school in the year before tube placement?
   1. yes, daycare
   2. yes, school
   3. yes, daycare and school
   4. no
8. Did your child take swimming lessons before the most recent tympanostomy tube(s) placement, or did he/she often swim in a pool, lake or sea?
   1. yes
   2. no
      1. did he/she regularly dip his/her head under water when bathing?
         - yes
         - no
9. Did your child use earplugs while swimming or bathing?
   1. yes, during swimming
   2. yes, during bathing
   3. yes, during both swimming and bathing
   4. no
10. Did your child use a dummy/pacifier in the year before tube placement?
    1. yes
    2. no

**About your family**

1. Does your son/daughter have any siblings and if so, how many?
   1. [number] older brothers/sisters
   2. [number] younger brothers/sisters
   3. no brothers or sisters
2. Do any other family members have a history of middle ear problems (many acute middle ear infections, or reduced hearing caused by fluid behind the eardrum)?
   1. his/her parent(s)
      1. yes
      2. no
   2. his/her brother(s)/sister(s)
      1. yes
      2. no
      3. not applicable
3. Did a physician ever diagnose asthma, hay fever, (atopic) eczema or a food allergy in any of his/her family members?
   1. his/her parent(s)
      1. yes
      2. no
   2. brother(s)/sister(s)
      1. yes
      2. no
      3. not applicable
4. At the time of the tube placement, did anyone smoke inside his/her home?
   1. yes
      1. in total, how many cigarettes/cigars were smoked inside his/her home each day?
         - number
   2. no
5. What is the highest level of education that his/her father has achieved?
   1. none
   2. primary school
   3. lower secondary school (VMBO, VBO, MAVO, LBO, LTS, VGLO)
   4. higher secondary school (MMS, HBS, HAVO, VWO)
   5. vocational school (MBO, MTS, MEAO)
   6. higher education (HBO, HTS, university)

1. What is the highest level of education that his/her mother has achieved?
   1. none
   2. primary school
   3. lower secondary school (VMBO, VBO, MAVO, LBO, LTS, VGLO)
   4. higher secondary school (MMS, HBS, HAVO, VWO)
   5. vocational school (MBO, MTS, MEAO)
   6. higher education (HBO, HTS, university)
