# Supplementary material for: Parent-Reported Otorrhea in Children with Tympanostomy Tubes: Incidence and Predictors
Source: PLoS One. 2013 Jul 12;8(7):e69062. doi: 10.1371/journal.pone.0069062 (PMC3709928; doi:10.1371/journal.pone.0069062)

Appendix S2

Timeline of study to determine the incidence of tympanostomy tube otorrhea and its predictors in children

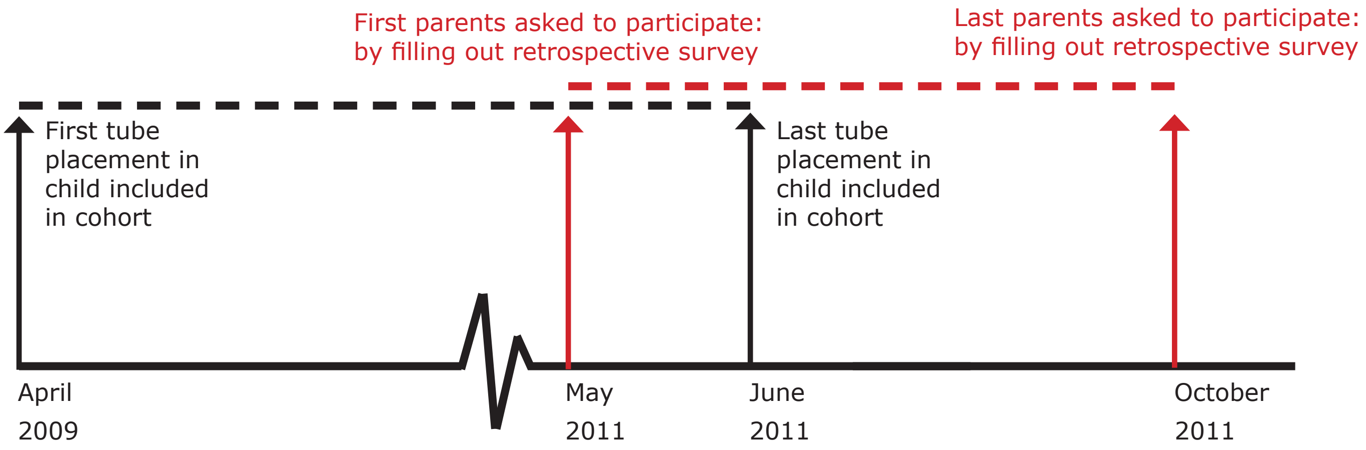

Supplement: Appendix S2 — (PDF) [file pone.0069062.s002.pdf]
